# High-trans fatty acid and high-sugar diets can cause mice with non-alcoholic steatohepatitis with liver fibrosis and potential pathogenesis

**DOI:** 10.1186/s12986-020-00462-y

**Published:** 2020-05-26

**Authors:** Xin Xin, Bei-Yu Cai, Cheng Chen, Hua-Jie Tian, Xin Wang, Yi-Yang Hu, Qin Feng

**Affiliations:** 1 Shuguang Hospital affiliated to Shanghai University of Traditional Chinese Medicine, 528 Zhangheng Road, Pudong New Area, Shanghai, 201203 China; 2 Shanghai Key Laboratory of Traditional Chinese Clinical Medicine, Shanghai, 201203 China; 3 Key Laboratory of Liver and Kidney Diseases, Shanghai University of Traditional Chinese Medicine, Ministry of Education, Shanghai, 201203 China

**Keywords:** NASH, Fibrosis, Animal model, Pathogenesis

## Abstract

**Background and aims:**

Even Non-alcoholic steatohepatitis (NASH) has been becoming the key role in process of liver fibrosis or cirrhosis, no any NASH involving liver fibrosis mice model which consistent with the mechanisms of fatty acid and glucose metabolism disorder was widely accepted. Here, we established a mouse model of nonalcoholic steatohepatitis (NASH) with liver fibrosis using a high-fat, high-carbohydrate diet (HFHC) and analyzed the potential pathogenesis using a transcriptome microarray.

**Methods:**

Fifty mice were stratified by weight and randomly divided into the HFHC model and control (Con) groups. Ten mice were sacrificed at the beginning of the experiments, 10 mice of HFHC and Con group were euthanized at the end of 20 and 30 weeks. The following analyses were performed: biochemical analysis; histological assessment; evaluation of hepatic type I collagen (Col-I), α-smooth muscle actin (α-SMA) and transforming growth factor-β1 (TGF-β1) protein and mRNA expression levels; and transcriptomic gene chip analysis.

**Results:**

Compared with the Con group at each time point, the body weight and liver wet weight of the HFHC model group of mice were significantly higher. At 30th weeks, alanine aminotransferase (ALT), aspartate aminotransferase (AST), fasting blood glucose (FBG) and fasting insulin (FINS) levels or activities and the triglyceride (TG) and hydroxyproline (HYP) content in the HFHC model group were significantly elevated. Severe steatosis was present in the liver tissues contributed from the HFHC group of mice. Typically, substantial perisinusoidal fibrosis with a cage-like structure and bridging formations were observed in the mice liver in HFHC group. Col-I, α-SMA and TGF-β1 protein and mRNA expression levels in liver tissues of HFHC mice dramatically increased over time. Compared with the Con group, the HFHC group had 151 differentially expressed genes that were involved in 41 signaling pathways.

**Conclusions:**

After keeping 30 weeks HFHC diet treatment, the mice exhibited substantial liver fibrosis, hepatic steatosis, ballooning degeneration and inflammation. Basing on the transcriptome microarray assays, the experimental NASH involving liver fibrosis potentially related to dramatically changed ECM-receptor interaction, Toll-like receptor signaling and other signaling pathways.

## Key points

- The incidence of fibrotic diseases is high, and there are no ideal therapeutic drugs or an ideal animal model to aid in drug development.
- By feeding mice a high-fat, high-sugar diet for 30 weeks, a nonalcoholic fatty hepatitis fibrosis mouse model was successfully established.
- The liver of this model mouse was genetically analyzed and screened to predict possible pathogenesis.

## Introduction

Nonalcoholic fatty liver disease (NAFLD) has become a common chronic liver disease worldwide. The disease spectrum of NAFLD includes nonalcoholic simple fatty liver (NAFL), nonalcoholic steatohepatitis (NASH), fatty liver fibrosis and liver cirrhosis [1]. Previous studies have shown that liver fibrosis is the most important predictive indicator affecting NAFLD prognosis, and fibrotic staging is also closely associated with long-term total mortality, liver transplant and liver-related events [2].

Animal models, especially mouse model, play important roles in investigating disease pathogenesis, assessing diagnostic methods and searching for effective preventive and therapeutic drugs. Establishment of appropriate animal models is important for understanding the pathogenesis of liver fibrosis in patients with NASH, and the following of drug efficacy assessment and pharmacological mechanisms investigation. Additionally, ideal NASH with fibrosis animal models would not only recapitulate the pathological changes in liver fibrosis in human NASH but also have other disease symptoms of metabolic syndrome, including obesity, hyperlipidemia and insulin resistance, which better simulate the background status of systemic metabolic disorders at human NASH onset. Currently, ideal animal models for fatty liver fibrosis are still rare (discussed below). A diet rich in saturated fatty acids and fructose is associated with the development of NASH as well as obesity in humans. A simple high-fat or high-carbohydrate diet can lead to inflammatory steatosis in the liver tissue of animals but fails in producing significant fibrosis. Attractively, Kohli et al. [3] announced successfully induced NASH-related liver fibrosis by using a mice model treated by a combination high-fat, high-carbohydrate (HFHC) diet for 16 weeks. However, according to their results, liver fibrosis was present in only 50% of the mice, and all of the fibrosis only stay at the F1 or F2 stage, without any mouse reaching to the F3 or F4 fibrotic stage [3]. Because only early-stage fatty liver fibrosis was observed in this animal model, this model is not ideal enough for assessment of the efficacy of antifibrosis treatments. Liver fibrosis in patients with NASH significantly prolongs disease duration, which is an important risk factor for the onset of liver fibrosis in patients with fatty liver disease. Therefore, based on the study by previous research [3], our current study extended the HFHC diet duration to 20, even 30 weeks with the same formulations used in the Kohli et al. [3] study. As we expected, we dynamically achieved successful liver fibrosis model rates and much more developed as well as serious fibrosis stage in this model. After this, we further analyzed the underlying molecular pathogenesis of NASH combined with fibrosis using transcriptome microarray analysis for providing an appropriate animal model for the following drug efficacy assessments in the future.

Herein, we are aim to develop a NASH model that generates significant hepatic fibrosis in non-genetically modified mice induced by a combined diet.

## Materials and methods

### Materials

#### Animals

Fifty 6-week-old, wild-type, male C57BL/6 SPF mice with a body mass of 20–22 g were purchased from Shanghai SLAC Laboratory Animal Co., Ltd. and kept, modeled and observed in a barrier-protected animal room in the animal research center at Shanghai University of Traditional Chinese Medicine. During model establishment, experimental animals had free access to food and water (SYXK (Shanghai) 2014–0008).

#### Diet

The diet for the HFHC model group was a 58 kcal% fat w/sucrose Surwit Diet (D12331, lot number: 17082101A10, Research Diets, USA), and the diet for the control (Con) group was an 11 kcal% fat w/cornstarch Surwit Diet (D12328, lot number: 17100212A4, Research Diets, USA). Fructose and sucrose (F0001/S0001) were purchased from Trophic Animal Feed High-Tech Co., Ltd., China.

### Methods

#### Animal grouping and model establishment

All animal experiment protocols were approved by Animal Experimental Ethics Committee of Shanghai University of Traditional Chinese Medicine. C57Bl/6 mice were housed in the animal research center at Shanghai University of Traditional Chinese Medicine. After 1 week of acclimation, the mice were randomly divided into the Con group (*n* = 30) and the HFHC model group (*n* = 20) according to their body weights. Mice in the HFHC model group were given free access to a high-fat (58% kcal fat, 25% kcal carbohydrate and 17% kcal protein) and high-carbohydrate (drinking water: 42 g/L, 55% fructose and 45% sucrose) diet, and the mice were equally divided into time points of 20 (HFHC 20w) and 30 weeks (HFHC 30w), with 10 mice at each time point. The Con group mice were fed a corresponding low-fat diet (10.5% fat, 73.1% kcal carbohydrate and 16.4% kcal protein) and normal drinking water; these mice were also equally divided into time points of 0 (Con 0w), 20 (Con 20w) and 30 weeks (Con 30w), with 10 mice at each time point.

#### Specimen collection

At the end of weeks 0, 20 and 30, the mice were fasted for 12 h and anesthetized with 2% pentobarbital sodium at a dose of 3 mL/kg via intraperitoneal injection. The eyeballs were removed, and 1 mL of blood was collected from each mouse. A portion of liver tissue was extracted from the same lobe and position in each mouse and fixed in 10% neutral buffered formalin solution.

#### Biochemical analysis

1) Hepatic enzymatic changes: Serum alanine aminotransferase (ALT) and serum aspartate aminotransferase (AST) were measured using ALT and AST assay kits (Lot number 20180628, Nanjing Jiancheng Bioengineering Institute, Nanjing, China). 2) Glucose metabolism: Following 12 h of fasting, approximately 3 μL of blood was collected from the tail vein, and fasting blood glucose (FBG) was measured using a Roche blood glucose meter (Roche diagnostic GmbH, Germany). Fasting insulin (FINS) levels in the serum of mice from each group were measured using an enzyme-linked immunosorbent assay (ELISA) kit (Ultra Sensitive Mouse Insulin ELISA Kit, lot number: 90080, Crystal Chem, USA). The homeostatic model assessment-insulin resistance (HOMA-IR) index was calculated with the following formula: FBG (mM) x FIN (IU/L)/22.5. 4) Liver triglyceride (TG) content was measured using a kit (Lot number: 2018080029, Dong’ou Diagnostic Products Co. Ltd., Zhejiang, China). 5) Liver hydroxyproline (HYP) content was measured using an HYP assay kit (lot number: 20180630, Nanjing Jiancheng Bioengineering Institute, Nanjing, China).

#### Hematoxylin and eosin (HE) and Sirius red staining

Fixed liver tissue was dehydrated and embedded using a tissue processor (Leica ASP300) and paraffin embedding station (Leica EG1160). Then, the sections were stained using an HE staining kit (lot number 20180530, Nanjing Jiancheng Bioengineering) and a Sirius red staining kit (lot number 20180528, Nanjing Jiancheng Bioengineering).

#### Oil red O staining

Liver tissue was fixed in liquid nitrogen, embedded in ornithine carbamoyl transferase (OCT) medium and sectioned at − 20 °C at a thickness of 10 μm. The sections were stained using an Oil red O staining kit (lot number 20180528, Nanjing Jiancheng Bioengineering).

#### Western blot analysis

Antibodies targeting the following proteins were used in this study: α-smooth muscle actin (α-SMA, ab5694, 1:1000); hepatic type I collagen (Col-I, ab34710, 1:1000); and transforming growth factor-β1 (TGF-β1, Abcam, 1:1000). Glyceraldehyde-3-phosphate dehydrogenase (GAPDH, proteintech,10,494–1-AP,1:10000) protein expression was used as an internal control.

#### Immunohistochemistry (IHC)

Samples were first incubated in anti-α-SMA antibody at a dilution of 1:100 (α-SMA, 1:100, catalog number: ab5694, USA) overnight at 4 °C and then incubated with primary antibody at a dilution of 1:250. Some liver sections were also stained with anti-Col-I antibody (Col-I, Abcam, 1:100, ab34710, USA) using the same method.

#### Reverse transcription polymerase chain reaction (RT-PCR)

RNA extraction: RNA was extracted using a UNIQ-10 pillar Trizol total RNA extraction kit (catalog number: E928KA9723, Sangon Biotech, Shanghai). The relative quantity (RQ) values of the PCR products were subjected to analysis using the △△CT method to assess the mRNA expression levels of Col-1, α-SMA, TGF-β1, Col-4 and Smad3.

#### Library preparation and Illumina HiSeq X ten sequencing

Libraries were size selected for cDNA target fragments of 200–300 bp on 2% Low Range Ultra Agarose, followed by PCR amplification using Phusion DNA polymerase (NEB) for 15 PCR cycles. After quantification with a TBS380 fluorometer, the paired-end RNA-seq sequencing library was sequenced with an Illumina HiSeq X Ten sequencing system (2 × 150 bp read length). The data were analyzed using the free online Majorbio I-Sanger Cloud Platform (www.i-sanger.com).

#### Statistical analysis

Statistical analysis of database data was performed using SPSS 22.0 software for Mac OS. The measurement data in the statistical description are indicated by S and refer to the count data. When the normality and homogeneity of the variance were satisfied, a t test was applied for comparison of the two groups. The comparison of the hierarchical grouping data was calculated via Radit analysis.

## Results

### Changes in food and water intake, body mass and liver wet weight levels between each group

#### Liver appearance in mice from each group

**Fig. 1:**
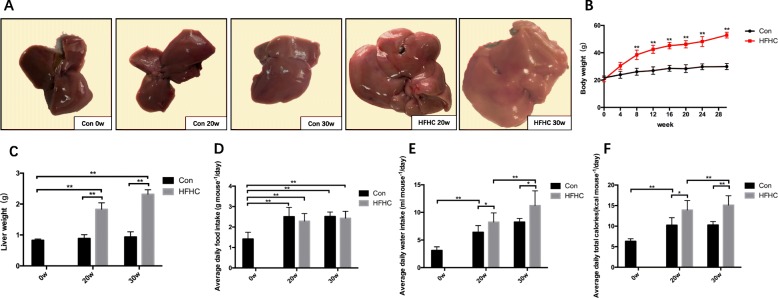
General conditions of the mice in each group. **a** Changes in liver morphology of the mice in each group. **b** Changes in body weight of the mice in each group. **c** Changes in liver wet weight of the mice in each group. **d-f** Changes in average food intake, water intake and total caloric intake of the mice in each group. Significant differences are indicated as follows: *P < 0.05, **P < 0.01

Grossly, the livers from mice in the Con group were dark red and soft. The livers from mice in the HFHC group showed different degrees of yellow at different time points and looked full and blunt; the sections were greasy, and the texture became tough. At 30 weeks, some livers from HFHC mice had yellowish white focal fat deposition in their livers (Fig. 1a).

#### Changes in average food intake, water intake and liver wet weight

No mice died during the experiment. The body mass of the mice in each group increased gradually over time (Fig. 1b). At 20 weeks, the body mass and liver wet weight in the HFHC group were significantly increased (*P* < 0.01). Compared with those of the Con group mice at 30w, the body mass and liver wet weight levels of the HFHC group mice at 30w were significantly higher (*P* < 0.01). Compared with those the HFHC group mice at 20w, the body mass and liver wet weight levels of the HFHC group mice at 30w were significantly higher (*P* < 0.05, *P* < 0.05) (Fig. 1c). The average food and water intake levels gradually increased over time, with no significant differences observed between the HFHC and Con groups at each time point (*P* > 0.05) (Fig. 1d-f).

### Enzymology and glucose metabolism status in each group

#### Changes in ALT and AST activities in each group

**Fig. 2:**
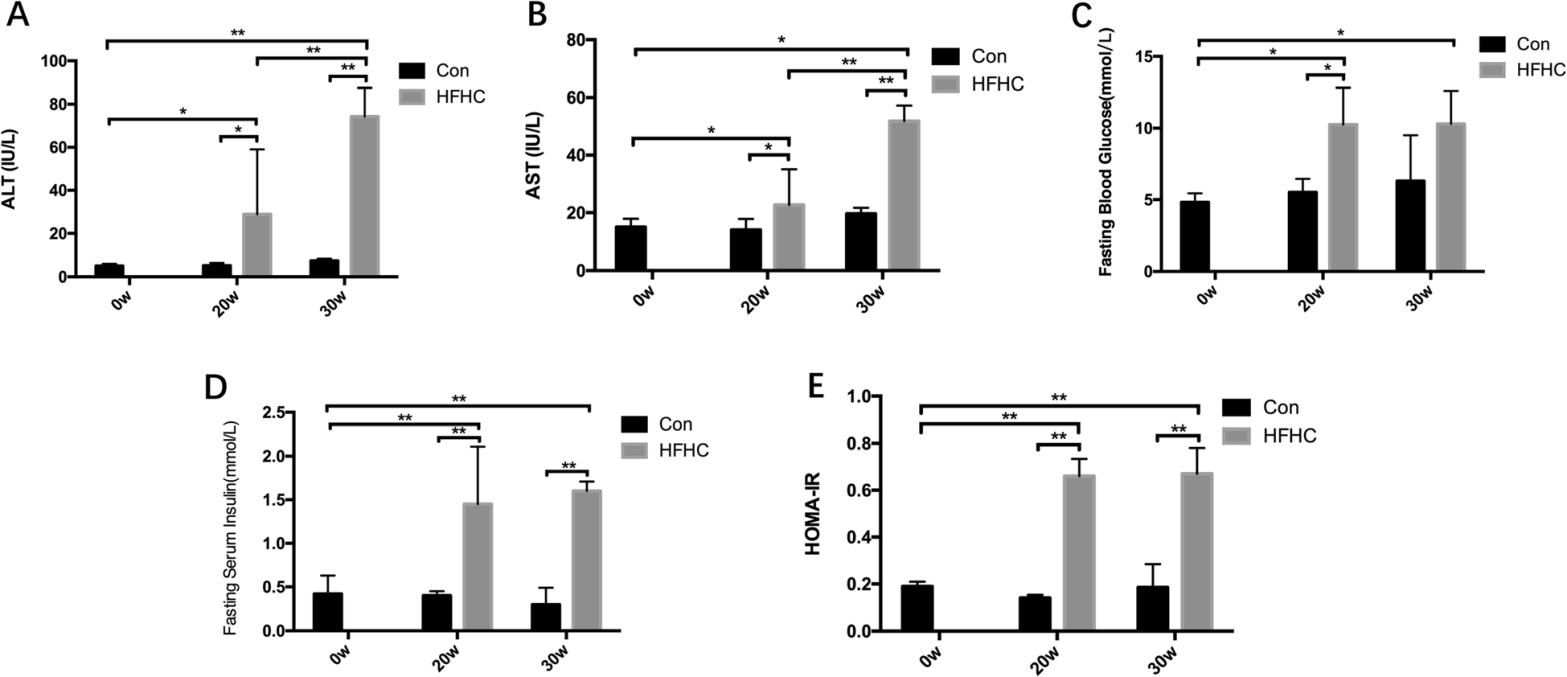
Enzymology and glucose metabolism status of the mice in each group. **a-e** Changes in serum ALT and AST activities, FBG, FINS and HOMA-IR of the mice from each group. Significant differences are indicated as follows: *P < 0.05, **P < 0.01

At 20 weeks, the serum ALT and AST activities in the HFHC group mice were significantly elevated (*P* < 0.01), and these activities increased progressively over time. At 30 weeks, the serum ALT and AST activities in the HFHC group mice were significantly higher than those in the Con group mice (*P* < 0.01) at 30w and the HFHC group mice at 20w (*P* < 0.01, *P* < 0.01) (Fig. 2a and b)*.*

#### Changes in FBG, FINS and HOMA-IR in each group

Compared with the Con group mice at 20w, the FBG and FINS levels in the HFHC group mice at 20w were significantly higher (*P* < 0.05, *P* < 0.01), and these levels increased progressively over time. At 30 weeks, the FBG and FINS levels in the HFHC group mice were elevated even more compared with those in Con group mice (*P* < 0.05, *P* < 0.01). Compared with the Con group mice at the same time points, the HOMA-IR indices in the HFHC group mice were significantly higher (*P* < 0.01, *P* < 0.05) (Fig. 2c-e).

### Liver steatosis and inflammation status of the mice in each group

#### Changes in liver TG content

**Fig. 3:**
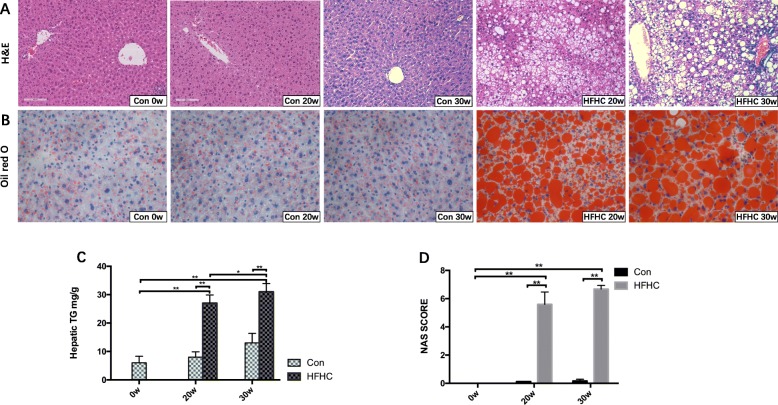
Liver steatosis and inflammation status of the mice in each group. **a** Liver tissue H&E staining (100x). **b** Oil red O staining (400x). **c** Changes in the liver TG contents of the mice in each group. **d** Changes in the NAS SCORE of the mice in each group. Significant differences are indicated as follows: *P < 0.05, **P < 0.01

The TG liver content increased gradually over time in both mouse groups. At 20 weeks, the TG liver content in the HFHC group was significantly elevated compared with that in the Con group (*P* < 0.01). Similarly, at 30 weeks, the TG liver content in the HFHC group was significantly elevated compared with that in the Con group (*P* < 0.01). Additionally, the TG content in the HFHC group at 30w was significantly higher than that at 20w (*P* < 0.05) (Fig. 3c)*.*

#### H&E staining

Under a light microscope, H&E staining of the liver tissue showed that hepatic steatosis affected the entire liver lobule, with substantial inflammatory cell infiltration and scattered necrosis. The HFHC mice at 30w showed ballooning degeneration of hepatocytes and inflammatory foci in the hepatic lobule. The NAFLD activity scores (NAS) ranged from 4 to 8, among which 80% were higher than 5, meeting the NASH diagnosis criteria, and 40% were close to 8 (Fig. 3a and d)*.*

#### Oil red O staining

Oil red O staining showed that both macrovesicular and microvesicular steatosis were present in the liver tissues from mice in the HFHC group at 20w, ranging from 33 to 66%. The area, density and intensity of Oil red O staining in liver tissue from the HFHC group increased gradually over time. At 30 weeks, severe macrovesicular and microvesicular steatosis was present in the liver tissues from the HFHC group, ranging from 66 to 99% (Fig. 3b)*.*

### Collagen deposition and hepatic stellate cell (HSC) activation status in liver tissue from mice in each group

#### HYP content

**Fig. 4:**
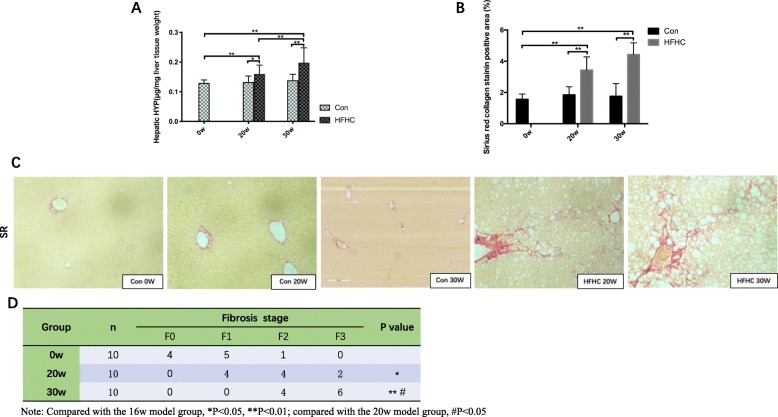
Collagen deposition in the liver tissue of the mice from each group (Pathology). **a** HYP content. **b** Liver fibrosis areas with positive collagen levels by semiquantitative analysis of Sirius red staining. **c** Sirius red staining (200×). **d** Liver fibrosis stage. Significant differences are indicated as follows: *, # *P* < 0.05, **P < 0.01

The liver HYP content in the HFHC group increased gradually over time. Compared with that in the Con group at 20w, the liver HYP content in the HFHC group mice at 20w was significantly increased (*P* < 0.05). Similarly, the liver HYP content in the HFHC group at 30w was significantly elevated compared with that in the Con group at 30w (*P* < 0.01) and the HFHC group at 20w (*P* < 0.05) (Fig. 4a)*.*

#### Sirius red staining

Under a light microscope, Sirius red staining of the liver tissue showed that at 20 weeks, fibrosis in the perisinusoidal space was present in a star shape in the HFHC mouse livers, and the fibrosis stage ranged from F1-F2, with the majority (60%) at stage F2. At 30 weeks, there was substantial perisinusoidal fibrosis in the liver, the presence of cage-like structures and massive fibrous connective tissue hyperplasia, with some bridging formations. Semiquantitative analysis showed that at 30 weeks, the collagen level in the HFHC group was significantly higher than that in the Con group (*P* < 0.01), and more than half of the fibrosis was at the F2-F3 stage (Fig. 4b-d)*.*

#### Changes in the col-I, α-SMA and TGF-β1 protein expression levels in liver tissues from mice in each group

**Fig. 5:**
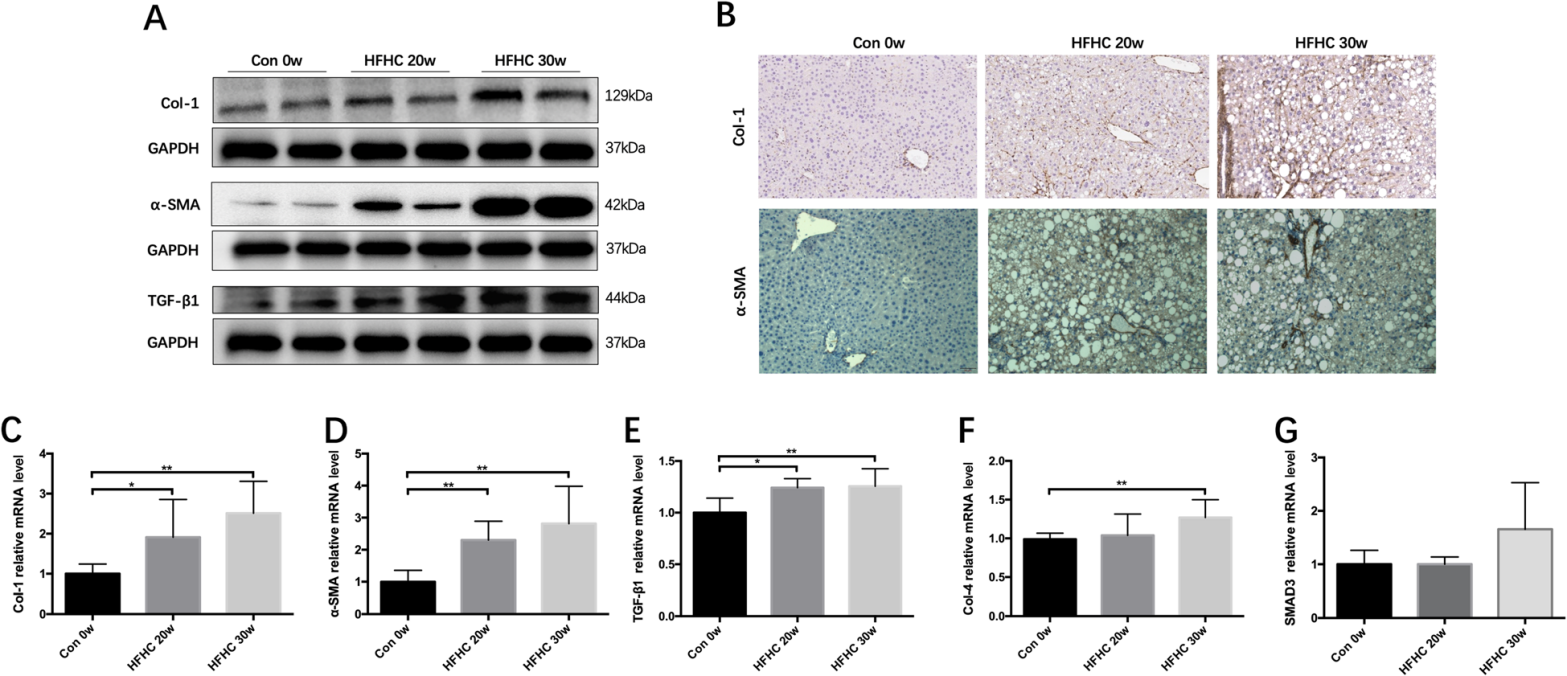
Collagen deposition and HSC activation status in the liver tissue of the mice from each group (protein and gene expression). **a** Col-1, α-SMA and TGF-β1 protein expression levels in liver tissue. **b** IHC of Col-1 and α-SMA in liver tissue. **c-g** The mRNA expression levels of Col-1, α-SMA, TGF-β1, Col-4 and Smad3 in liver tissue. Significant differences are indicated as follows: *P < 0.05, **P < 0.01

Western blot analysis showed that the Col-I, α-SMA and TGF-β1 protein expression levels in the HFHC group livers at 20w and 30w were significantly higher than those in Con group livers at 0w, and the expression levels increased over time (*P* < 0.01, *P* < 0.01, *P* < 0.05) (Fig. 5a)*.*

#### IHC of Col-1 and α-SMA in the mouse livers in each group

This α-SMA-positive staining was significantly enhanced in the fibrotic liver septum in the HFHC group at 20w and 30w and was mainly located in the thick and dense fibrotic septum. The positive staining area increased over time. Similarly, Col-I positive staining was present only in the blood vessel walls in the Con group at 0w. This Col-I positive staining was significantly enhanced in the fibrotic liver septum in the HFHC group at 20w and 30w and was mainly located in the perisinusoidal hepatocyte membranes and fibrotic septum. The positive staining area increased over time (Fig. 5b)*.*

#### Col-I, Col-4, α-SMA, TGF-β1 and Smad3 mRNA expression levels in mice in each group

Compared with those in the Con group livers at 0w, the Col-I, TGF-β1 and α-SMA mRNA expression levels in the HFHC group livers at 20w were significantly increased (*P* < 0.05, *P* < 0.05 and *P* < 0.01). At 30 weeks, the Col-I, Col-4, α-SMA and TGF-β1 mRNA levels in the HFHC group livers were significantly increased (*P* < 0.01), and there was a trend toward increased Smad3 mRNA levels, although the difference was not significant (Fig. 5c-g)*.*

### Transcriptome analysis of differential gene expression between the HFHC group and con group

#### Model group and normal group differential gene clustering analysis heat map

**Fig. 6:**
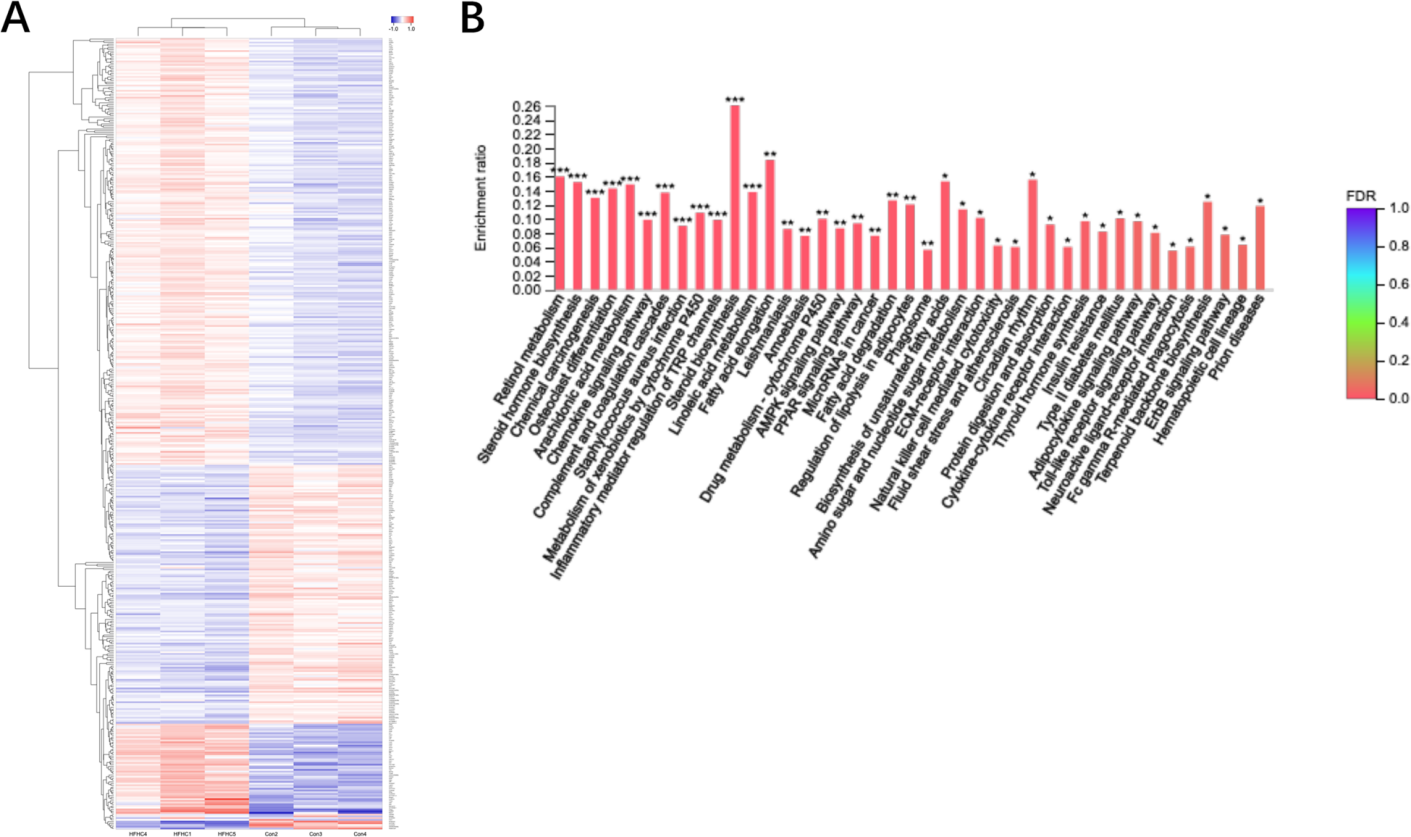
Transcriptome gene chip analysis. **a** Model group and normal group difference gene clustering analysis heat map. **b** Differences between the model group and the normal group KEGG enrichment analysis. Note: The abscissa indicates the name of the path; the ordinate indicates the enrichment rate, and the formula is as follows: (Enrichment Ratio = Sample Number/Background Number). Color indicates a significant enrichment *P* value i.e., the deeper the color indicates the passage significantly enriched, where ***P < 0.001, **P < 0.01, *P < 0.05. The side color gradient represents the P value size

The genes differentially expressed between the normal group and model group were selected (*P* < 0.05), and a heat map analysis was performed via cluster analysis, as shown in the following figure. The three samples in the normal group and the model group were naturally clustered, and the expression levels of the genes were similar. However, the difference in gene expression between the two groups was significant (Fig. 6a).

#### Differentially expressed genes between the model group and normal group

There were 151 differentially expressed genes with Log2FC ≥ 2 and P < 0.05 between the model group and the normal group, including 104 upregulated genes (Table 1) and 47 downregulated genes (Table 2).

**Table 1:** Differentially upregulated genes in the model group and the control group with Log2FC ≥ 2 and *P* < 0.05

Gene name	Gene description	Con (Mean)	HFHC (Mean)	FC (HFHC/Con)	Log2FC (HFHC/Con)	Padjust	Regulate
Sprr1a	Small Proline-rich Protein 1A	0.00	4.59	431.01	8.75	0.0132	up
Cyp2b10	Cytochrome P450, family 2, subfamily b, polypeptide 10	0.10	25.61	274.92	8.10	0.0163	up
Cidea	Cell death-inducing DNA fragmentation factor, alpha subunit-like effector A	0.22	46.42	217.00	7.76	0.0001	up
Mas1	MAS1 oncogene	0.00	0.57	118.42	6.89	0.0156	up
Xlr4a	X-linked lymphocyte-regulated 4A	0.02	3.93	82.35	6.36	0.0330	up
Chil1	Chitinase-like 1	0.03	1.22	79.61	6.31	0.0043	up
Ttc39aos1	Ttc39a opposite strand RNA 1	0.00	1.46	67.28	6.07	0.0409	up
Cyp2b9	Cytochrome P450, family 2, subfamily b, polypeptide 9	1.56	123.01	62.17	5.96	0.0044	up
Prtn3	Proteinase 3	0.00	0.78	59.71	5.90	0.0444	up
Cyp2b13	Cytochrome P450, family 2, subfamily b, polypeptide 13	0.03	1.66	56.33	5.82	0.0103	up
Cfd	Complement factor D (adipsin)	0.23	12.86	52.08	5.70	0.0015	up
Orm3	Orosomucoid 3	0.27	7.06	25.29	4.66	0.0082	up
Nat8f7	N-acetyltransferase 8 (GCN5-related) family member 7	0.06	2.18	25.21	4.66	0.0184	up
Pls1	Plastin 1 (I-isoform)	0.14	0.69	21.32	4.41	0.0161	up
Ciart	Circadian associated repressor of transcription	0.27	5.84	20.40	4.35	0.0186	up

**Table 2:** Differentially downregulated genes in the model group and the control group with Log2FC ≤ − 2 and *P* < 0.05

Gene name	Gene description	Con (Mean)	HFHC (Mean)	FC (HFHC/Con)	Log2FC (HFHC/Con)	Padjust	Regulate
Adra2a	Adrenergic receptor, alpha 2a	0.47	0.02	0.04	−4.70	0.0240	Down
Serpina1e	Serine (or cysteine) peptidase inhibitor, clade A, member 1E	2355.30	94.32	0.04	−4.51	0.0052	Down
Lepr	Leptin receptor	7.01	0.54	0.06	−4.04	0.0043	Down
Cspg5	Chondroitin sulfate Proteoglycan 5	1.11	0.07	0.06	−3.96	0.0046	Down
Enho	Energy homeostasis associated	13.42	1.12	0.09	−3.52	0.0025	Down
Slc30a3	Solute carrier family 30 (zinc transporter), member 3	0.94	0.08	0.09	−3.49	0.0051	Down
Unc79	Unc-79 homolog (*C. elegans*)	1.15	0.14	0.13	−3.00	0.0181	Down
Gtpbp4-Ps1	GTP binding Protein 4, Pseudogene 1	54.76	5.16	0.13	−2.97	0.0083	Down
Pitx3	Paired-like homeodomain transcription factor 3	6.66	0.52	0.14	−2.87	0.0033	Down
Slc13a5	Solute carrier family 13 (sodium-dependent citrate transporter), member 5	1.25	0.20	0.14	−2.86	0.0149	Down
Fam198a	Family with sequence similarity 198, member A	5.43	0.78	0.14	−2.80	0.0052	Down
Pdk4	Pyruvate dehydrogenase kinase, isoenzyme 4	33.33	5.43	0.15	−2.74	0.0041	Down
Asns	Asparagine synthetase	1.17	0.40	0.16	−2.68	0.0161	Down
PPP1r3g	Protein phosphatase 1, regulatory (inhibitor) subunit 3G	2.54	0.41	0.16	−2.66	0.0181	Down
Fst	Follistatin	1.47	0.24	0.17	−2.60	0.0056	Down

#### Signal-regulated pathways associated with the genes differentially expressed between the model group and the normal group

The 151 differentially expressed genes between the model group and the normal group with a fold change ≥2 according to Log2FC values were analyzed via KEGG PATHWAY analysis using R script. When the corrected *P* value (P adjust) was < 0.05, the KEGG PATHWAY function was considered to show significant enrichment. The results showed that the above 151 differentially expressed genes were involved in 41 signaling pathways, suggesting that the 41 signaling pathways are significant in the high-trans fatty acid, high-glucose diet-induced NAFLD model (Fig. 6b, Table 3).

**Table 3:** KEGG signaling pathways identified in genes that are differentially expressed between the model group and the control group

Pathway id	Description	Ratio_in_study	Ratio_in_pop	Percent	***P*** value
Map00830	Retinol metabolism	23/665	143/22793	16.08%	1.4977E-08
Map00140	Steroid hormone biosynthesis	22/665	144/22793	15.28%	2.6183E-08
Map05204	Chemical carcinogenesis	23/665	176/22793	13.07%	1.3555E-07
Map00590	Arachidonic acid metabolism	19/665	127/22793	14.96%	2.8598E-07
Map04062	Chemokine signaling pathway	24/665	241/22793	9.96%	8.3868E-06
Map00980	Metabolism of xenobiotics by cytochrome P450	15/665	137/22793	10.95%	0.00035448
Map04750	Inflammatory mediator regulation of TRP channels	16/665	161/22793	9.94%	0.00056316
Map00100	Steroid biosynthesis	6/665	23/22793	26.09%	0.00098829
Map00591	Linoleic acid metabolism	10/665	72/22793	13.89%	0.00099817
Map00062	Fatty acid elongation	7/665	38/22793	18.42%	0.00210167
Map00982	Drug metabolism-cytochrome P450	12/665	119/22793	10.08%	0.00324254
Map04152	AMPK signaling pathway	14/665	160/22793	8.75%	0.00416512
Map03320	PPAR signaling pathway	12/665	127/22793	9.45%	0.00529036
Map00071	Fatty acid degradation	8/665	63/22793	12.70%	0.00646651
Map04923	Regulation of lipolysis in adipocytes	8/665	66/22793	12.12%	0.00846127
Map04145	Phagosome	27/665	474/22793	5.70%	0.00990327
Map01040	Biosynthesis of unsaturated fatty acids	6/665	39/22793	15.38%	0.01027708
Map00520	Amino sugar and nucleotide sugar metabolism	8/665	70/22793	11.43%	0.01099148
Map04512	ECM-receptor interaction	9/665	88/22793	10.23%	0.01163767
Map04650	Natural killer cell mediated cytotoxicity	19/665	302/22793	6.29%	0.01575303
Map04974	Protein digestion and absorption	9/665	97/22793	9.28%	0.01992439
Map04060	Cytokine-cytokine receptor interaction	19/665	311/22793	6.11%	0.02042395
Map04918	Thyroid hormone synthesis	8/665	82/22793	9.76%	0.02369879
Map04931	Insulin resistance	10/665	121/22793	8.26%	0.02475499
Map04930	Type II diabetes mellitus	7/665	69/22793	10.14%	0.03251649
Map04920	Adipocytokine signaling pathway	7/665	72/22793	9.72%	0.0399927
Map04620	Toll-like receptor signaling pathway	9/665	111/22793	8.11%	0.04083019
Map04080	Neuroactive ligand-receptor interaction	19/665	341/22793	5.57%	0.04192223
Map04666	Fc gamma R-mediated phagocytosis	15/665	244/22793	6.15%	0.0422101
Map00900	Terpenoid backbone biosynthesis	5/665	40/22793	12.50%	0.04231822
Map04012	Erbb signaling pathway	9/665	115/22793	7.83%	0.04606007

## Discussion

NAFLD has become a common chronic liver disease worldwide, and its disease spectrum includes NAFL, NASH, fatty liver fibrosis and liver cirrhosis. Currently, NAFLD affects 30% of the population in western countries and 15–40% in China. More than 50% of obese individuals have NAFLD, and 30–42% of severely obese NASH patients have significant liver fibrosis [1]. A recent study showed that persistent liver injury progresses to liver fibrosis and liver cirrhosis in approximately 40% of NASH patients. Within a decade, the large population of NASH patients could lead to a high incidence of fatty liver fibrosis and liver cirrhosis. Long-term follow-up studies have shown that liver fibrosis is the most important predictive indicator affecting NAFLD prognosis [4, 5].

Selection of an appropriate animal models is critical for investigations into the pathogenesis of fatty liver fibrosis as well as the therapeutic drugs development [6]. An ideal animal model would not only recapitulate the pathological changes associated with liver fibrosis in human NASH but also exhibit other metabolic syndrome disease symptoms, including obesity, hyperlipidemia and insulin resistance, which better simulate the background status of systemic metabolic disorders present at the onset of human NASH. Significant progress has been made in the establishment of NAFLD animal models, and in recent years, simple fatty liver animal models have been successfully established. However, currently, ideal animal models for fatty liver fibrosis research still keep less but urgency. At present, animal models used to study NAFLD primarily include the following: gene knockout/mutation models, animal models induced by chemical toxins, drugs or nutrition deficiency, high-fat diet models, and composite models. Generally, animal models established by genetic mutations or deletions have impaired fatty acid metabolism, leading to spontaneous fatty liver formation accompanied by relevant metabolic syndrome symptoms, which may result in hepatocellular carcinoma (HCC) instead of leading to NASH or the natural progressive of fibrosis. As a result, those genetic models are only suitable for the investigation of specific genetic corresponding pathogenesis in NAFLD [7]. Among the chemical toxin or drug-induced animal models, the CCl4-induced model is the earliest developed and is the most widely used. This model is characterized by a short induction duration, with significant pathological alteration in liver tissue. However, the greatest limitation of the CCl4-induced model is that the animals do not become obese or develop insulin resistant, which differs greatly from the pathophysiological characteristics in metabolic disorder induced NAFLD patients [8]. Another typical model because of nutrient deficiency is the model induced by the methionine-choline-deficient (MCD) diet. This model shows the typical NASH histological changes but is accompanied by unwanted weight loss and the absence of metabolic syndrome [9]. Taken together, NASH involving progressive hepatic fibrosis animal model, which pathogenesis mainly correspond with nutritional metabolic disorder still keep required.

Increasing researches highlight that a diet rich in saturated fat and fructose should take major responsibility of the development of obesity and NASH in humans. Animals fed with a high-fat or high-carbohydrate diet can successfully induce inflammation and steatosis but fails to generate significant fibrosis in liver. HFHC diets may lead to significant liver fibrosis, inflammation, endoplasmic reticulum stress and apoptosis in fat cells in humans and animals [10]. Multiple clinical studies have confirmed that excessive Trans fatty acids (TFAs) consumption increases the probability of developing NASH and excessive TFA intake reduces the sensitivity of fat cells to insulin, which in turn increases the insulin consumption and the pancreatic load. This vicious cycle is highly leading to the incidence of type II diabetes in obese individuals [11]. Similarly, excessive TFA intake has been shown to induce liver steatosis by increasing fatty acid synthesis and inhibiting fatty acid oxidation [12]. Other studies have shown that feeding LDL receptor-knockout mice [13] and PPARα-knockout mice [14] a high-fat diet enriched in TFAs can accelerate NASH induction. Tetri et al. [15] pointed out under the circumstances of obesity and insulin resistance, mice fed fructose and TFAs developed NASH after 12 weeks, and the study further showed that TFAs caused liver injury under certain conditions. Additionally, Kondoh et al. [16] showed that the addition of elaidic acid, the main component of partially hydrogenated vegetable oils, to human liver cells led to apoptosis by activating caspase 3. All above studies explained that TFAs play a critical role in the progression of NAFLD to NASH combined with liver fibrosis. Currently, some researchers believe that a combination of TFAs and fructose induces oxidative stress and increases TNFα, IL-6, IL-1β and collagen-Iα expression levels, which partly explained the roles of TFA and fructose in liver fibrosis progression. Additionally, fructose metabolism leads to glyoxal formation and increases reactive oxygen species levels, which induces mitochondrial damage in liver cells. Fructose is also involved in liver steatosis and liver disease progression by promoting lipid synthesis, leptin resistance and the generation of long-lasting endotoxins [10]. However, it should be pointed out here that a simple high-fructose diet is insufficient to induce liver inflammation or fibrosis, and this diet reduces water intake in mice.

A combination of fructose and sucrose ensures normal water intake in experimental animals.

After systematic analysis, careful comparison, and in-depth study of the current mainly accepted research models, we cautiously decided to feed mice a high-fat (especially TFAs) diet plus an excessive amount of fructose and sucrose according to a study published in *Hepatology* [3]. Based on that study, we set different time points, extended the model establishment duration to 20 and 30 weeks. Importantly, we dynamically observed the pathological features of these mice model, including but not limited to liver steatosis, inflammation and collagen deposition. Thus, we established an ideal model that better recapitulates NASH with liver fibrosis and further contribute to the therapeutic drug efficacy assessments in the future.

Our study showed that following 30 weeks of a high-fat (saturated fat + TFAs) and high-carbohydrate (fructose + sucrose) diet the mice were fat and sluggish. Compared with the control group over the same time, the mice in the HFHC model group did not differ significantly in the amount of food as well as water intake. After 30 weeks HFHC feeding, the body weight of the HFHC group mice at 30w was increased by approximately 100% compared with that of the Con group mice at 30w and 20% compared with that of the HFHC group mice at 20w. The HYP content in the HFHC group was higher than that in the Con group at the same time points. At 20 weeks, 80% of the HFHC mice met the pathological diagnostic criteria of NASH, which was accompanied by perisinusoidal fibrosis. Liver pathological analysis showed that at 20 weeks, macrovesicular and microvesicular steatosis was present in the liver tissues from the HFHC group, ranging from 33 to 66%; inflammatory foci began to appear in the hepatic lobule; fibrosis in the perisinusoidal space was distributed in a star shape; and fibrosis staging ranged from F1–2, with the majority (60%) being at stage F2. At 20 weeks, 80% of the HFHC mice met the pathological diagnostic criteria for NASH, which was accompanied by perisinusoidal fibrosis. With the progress of the NASH, the liver tissues from the HFHC group at 30w showed more severe fibrosis and fat deposition. The presence of severe macrovesicular and microvesicular steatosis ranged from 66 to 99%, which was accompanied by ballooning degeneration in hepatocytes and inflammatory foci in the hepatic lobule. No surprisingly, the liver pathological scores of mice in the HFHC group at 30w were further elevated in an encouraging way. Sirius red staining showed substantial perisinusoidal fibrosis, the presence of cage-like fibrotic changes, and bridging formations in some of the fibrotic tissue. Semiquantitative analysis confirmed showed significantly higher collagen levels in the HFHC group at 30w. Additionally, more than 70% of the fibrosis was at stages F2-F3. α-SMA, Col-I and TGF-β1 protein and mRNA expression in the livers of the HFHC mice at 20w were significantly higher than those in the livers of the Con group mice at 20w. And significant increases in α-SMA-positive and Col-I-positive staining, which indicative of HSC activation and collagen deposition in the liver were observed following 20 weeks of a HFHC diet. What’s more, α-SMA, Col-I and TGF-β1 protein and mRNA levels were further stimulated in the livers of the HFHC mice at 30w. α-SMA- and Col-I-positive staining areas in the livers of the HFHC mice at 30w were also significantly increased with 10 weeks continuing HFHC feeding. At 30 weeks, the HFHC mice exhibited elevated serum ALT, AST and TG contents, which was accompanied by disrupted glucose metabolism—consistent with natural NASH progression. The HFHC mice at 30w also exhibited major symptoms of metabolic syndrome, indicating that this extended HFHC induction method successfully establishes an animal model including serious as well as progressive liver fibrosis in NASH.

Basing on the successful construction of this mouse model of NASH with liver fibrosis which mimic the pathogenesis and symptoms happened in patients, in order to better reveal the pathogenic mechanism of this model and lay the foundation of new drug development, we compared the gene expression levels the control group with the model group. Through analysis, we found 151 genes with Log2FC difference multiples greater than 2 and *P* < 0.05 in the high-fat and high-sugar diet group compared with the normal group and used the R script to perform KEGG PATHWAY enrichment analysis of the genes/transcripts in the gene set. A corrected *P* value < 0.05 was considered to indicate significant enrichment of the KEGG PATHWAY function. The results showed that the above 151 differentially expressed genes were involved in 41 signaling pathways, these are significant changed in the mouse NASH with fibrosis model. Based on their roles and pathophysiological significance, the 41 signaling pathways associated with these 151 differentially expressed genes revealed in the gene expression analysis likely play a potential role in the pathological characteristics and related pathogenesis of NASH with fibrosis in mice.

The results of this transcriptome analysis in the 30-week mice that were fed a high-trans fatty acid and high-sugar water diet revealed significant changes in the expression of 151 genes involved in 41 pathways. Thus, the following pathways highly correlated with the development of NAFLD: the ECM-receptor interaction pathway; the Toll-like receptor signaling pathway; cytokine-cytokine receptor interaction; the retinol metabolism pathway; steroid hormone biosynthesis; arachidonic acid metabolism; the chemokine signaling pathway; steroid biosynthesis; linoleum/linoleic acid metabolism; the adenosine 5′-monophosphate (AMP) signaling (AMPK signaling) pathway; the peroxisome proliferator-activated receptor signaling (PPAR signaling) pathway; fatty acid degradation; and regulation of lipolysis in adipocytes. Furthermore, liver fibrosis-related signaling pathways were particularly prominent.

## Conclusions

In summary, the mouse model of NASH and liver fibrosis described herein, which was induced by 30 weeks of a high-fat (saturated fat + TFAs) and high-carbohydrate (fructose + sucrose) diet, closely mimics NASH associated with liver fibrosis pathological process in patients. The high incidence of these pathological changes, high success rate of model establishment, and zero mortality rate make this model appropriate for investigating the underlying mechanisms of liver fibrosis within NASH and for drug efficacy assessments.

## Data Availability

All data generated or analyzed during this study are included in this published article.

## Abbreviations

NASH: Nonalcoholic steatohepatitis
NAFLD: Nonalcoholic fatty liver disease
HFHC: High-fat, high-carbohydrate diet
ND: Normal diet
TG: Triglyceride
IR: Insulin resistance

## References

[1] Diehl AM, Day C (2017). Cause, pathogenesis, and treatment of nonalcoholic steatohepatitis. N Engl J Med.

[2] Angulo P, Kleiner DE, Dam-Larsen S (2015). Liver fibrosis, but no other histologic features, is associated with long-term outcomes of patients with nonalcoholic fatty liver disease. Gastroenterology.

[3] Kohli R, Kirby M, Xanthakos SA (2010). High-fructose, medium chain trans fat diet induces liver fibrosis and elevates plasma coenzyme Q9 in a novel murine model of obesity and nonalcoholic steatohepatitis. Hepatology.

[4] Ekstedt M, Hagstrom H, Nasr P (2015). Fibrosis stage is the strongest predictor for disease-specific mortality in NAFLD after up to 33 years of follow-up. Hepatology.

[5] Hagstrom H, Nasr P, Ekstedt M (2017). Fibrosis stage but not NASH predicts mortality and time to development of severe liver disease in biopsy-proven NAFLD. J Hepatol.

[6] London RM, George J (2007). Pathogenesis of NASH: animal models. Clin Liver Dis.

[7] Anstee QM, Goldin RD (2006). Mouse models in non-alcoholic fatty liver disease and steatohepatitis research. Int J Exp Pathol.

[8] Kubota N, Kado S, Kano M (2013). A high-fat diet and multiple administration of carbon tetrachloride induces liver injury and pathological features associated with non-alcoholic steatohepatitis in mice. Clin Exp Pharmacol Physiol.

[9] George J, Pera N, Phung N, Leclercq I, Hou JY, Farrell G (2003). Lipid peroxidation, stellate cell activation and hepatic fibrogenesis in a rat model of chronic steatohepatitis. J Hepatol.

[10] Lee O, Bruce WR, Dong Q, Bruce J, Mehta R, O'Brien PJ (2009). Fructose and carbonyl metabolites as endogenous toxins. Chem Biol Interact.

[11] Jeyapal S, Putcha UK, Mullapudi VS (2018). Chronic consumption of fructose in combination with trans fatty acids but not with saturated fatty acids induces nonalcoholic steatohepatitis with fibrosis in rats. Eur J Nutr.

[12] Obara N, Fukushima K, Ueno Y (2010). Possible involvement and the mechanisms of excess trans-fatty acid consumption in severe NAFLD in mice. J Hepatol.

[13] Machado RM, Stefano JT, Oliveira CP (2010). Intake of trans fatty acids causes nonalcoholic steatohepatitis and reduces adipose tissue fat content. J Nutr.

[14] Hu X, Tanaka N, Guo R (2017). PPARalpha protects against trans-fatty-acid-containing diet-induced steatohepatitis. J Nutr Biochem.

[15] Tetri LH, Basaranoglu M, Brunt EM, Yerian LM, Neuschwander-Tetri BA (2008). Severe NAFLD with hepatic necroinflammatory changes in mice fed trans fats and a high-fructose corn syrup equivalent. Am J Physiol Gastrointest Liver Physiol.

[16] Kondoh Y, Kawada T, Urade R (2007). Activation of caspase 3 in HepG2 cells by elaidic acid (t18:1). Biochim Biophys Acta.

